# Predictive Validity of Image-Based Motivation-to-Eat Visual Analogue Scales in Normal Weight Children and Adolescents Aged 9–14 Years

**DOI:** 10.3390/nu14030636

**Published:** 2022-02-01

**Authors:** Leila Hammond, Olivia Morello, Michaela Kucab, Julia O. Totosy de Zepetnek, Jennifer J. Lee, Tarah Doheny, Nick Bellissimo

**Affiliations:** 1 School of Nutrition, Ryerson University, Toronto, ON M5B 2K3, Canada; leila.hammond@ryerson.ca (L.H.); olivia.morello@ryerson.ca (O.M.); michaela.kucab@ryerson.ca (M.K.); j585lee@ryerson.ca (J.J.L.); tarahdoheny@gmail.com (T.D.); 2 Faculty of Kinesiology and Health Studies, University of Regina, Regina, SK S4S 0A2, Canada; julia.totosy@uregina.ca

**Keywords:** motivation-to-eat, subjective appetite, food intake, children, adolescents, visual analogue scale

## Abstract

Paper-based motivation-to-eat visual analogue scales (VASs) developed for adults are widely used in the pediatric age range. The VAS is comprised of four domains: hunger, fullness, desire to eat, and prospective food consumption. The purpose of the present study was to determine agreement between the traditional paper-based VAS and a novel digital VAS (with and without images), as well as the novel digital VAS’s predictive validity for subsequent food intake (FI) in 9–14-year-old children and adolescents. Following an overnight fast and 3 h after consuming a standardized breakfast at home, children and adolescents (*n* = 17) completed three different VAS instruments (VAS_paper_, VAS_images_, VAS_no-images_) in a randomized order at five time-points: 0 min (baseline), 5 min (immediately after consuming a 147 kcal yogurt treatment), 20 min, 35 min (immediately before an ad libitum lunch), and 65 min (immediately post ad libitum lunch). All three instruments were comparable, as shown by low bias and limits of agreement on Bland–Altman plots, moderate to excellent intraclass correlation coefficients for all domains at all time-points (ICC = 0.72–0.98), and no differences between the incremental area under the curve for any of the domains. All three instruments also showed good predictive validity for subsequent FI, with the strongest relationship observed immediately before the ad libitum lunch (*p* = 0.56–0.63). There was no significant association between subjective thirst and water intake, except with VAS_no-images_ at baseline (r = 0.49, *p* = 0.046). In conclusion, the present study suggests that a novel image-based digital VAS evaluating motivation-to-eat is interchangeable with the traditional paper-based VAS, and provides good predictive validity for next-meal FI in 9–14-year-old normal weight children and adolescents.

## 1. Introduction

The most common approach to assess subjective appetite is to ask participants a series of questions relating to motivation-to-eat with the use of paper-based visual analogue scales (VASs) [1,2]. Originally developed by Hill and Blundell, the VAS assessing motivation-to-eat comprised six questions: ‘How strong is your desire to eat?’; ‘How hungry do you feel?’; ‘How full do you feel?’; ‘How much do you think you could eat?’; ‘Urge to eat’; and ‘Preoccupation with thoughts of food’ [2]. In recent years, variations of the original motivation-to-eat VAS have been used in appetite trials [3,4,5,6,7,8,9,10]. Overall, paper-based VASs assessing motivation-to-eat show good within-subject reliability and predictive ability for subsequent food intake in adults [11,12].

There has been a gradual shift away from traditional paper-based VASs towards digital VAS instruments [3,13,14,15]. Paper-based VASs are time consuming and are subject to potential human error, such as mismeasurement of subject ratings and transcription errors [16]. Digital VASs may avoid the limitations of paper-based VAS while remaining a relatively low-cost option. Earlier advances in digital VASs resulted in the development of the Electronic Appetite Ratings System (EARS) that has been used in various studies, primarily in adult populations [16]. More recently, the APPetite application has been compared to the traditional paper-based VAS in young adults in free-living settings [17]. Overall, this previous research has found that motivation-to-eat sensations are comparable between the paper and digital versions of VAS, including EARS and APPetite [17,18,19].

It has been reported that children lack the conceptual ability to operationalize and separate their feelings of appetite, as is required when motivation-to-eat VASs [originally designed for adults] are used [20]. The only study that has assessed the reproducibility and validity of motivation-to-eat VASs in children and adolescents found weak associations between motivation-to-eat VASs and test meal food intake (FI) in 9–14-year-old boys [21]. Picture-based motivation-to-eat VASs have since been developed for use in pediatric appetite trials as a means of increasing comprehension [22,23,24,25], though these VASs have not been used in older children and adolescents. There are no other published studies using digital picture-based VASs to assess motivation-to-eat in youth, nor are there any studies that have reported the validity or utility of VASs to predict subsequent FI in youth.

Establishing the predictive validity for FI of new digital VAS instruments in children and adolescents is important given the limitations of paper-based VASs and the limited research in this population. Therefore, the objectives of the present study were two-fold: (1) to evaluate the agreement between traditional paper-based VAS and a novel digital motivation-to-eat VAS (with and without images) in children and adolescents; and (2) to evaluate the predictive validity of a novel digital VAS (with and without images) for FI at an ad libitum meal in children and adolescents.

## 2. Materials and Methods

### 2.1. Participants

Boys and girls aged 9–14 years were recruited through community advertisements and by word-of-mouth. Inclusion criteria were habitual breakfast eaters who had a normal body mass index (BMI) according to the Centers for Disease Control [26], and who were born full term with a normal birth weight. Exclusion criteria were children with behavioral, learning, or developmental conditions, those who had any previously diagnosed medical conditions and/or who currently take medications, and those with allergies to test-day foods. Parents of potential participants were screened over the phone by a trained researcher to determine the eligibility of their child using a scripted questionnaire. Children that were eligible based on the information provided were invited to attend an in-person study information session at Ryerson University.

At the information session, written consent and assent were obtained from the parent and child, respectively. Participants were familiarized with the study protocol and test-day procedures, including how to use the test-day study instruments. Height was measured to the nearest 0.1 cm (Seca, Hamburg, Germany), body mass was measured in kilograms (COSMED USA Inc., Concord, CA, USA), and BMI percentile was calculated. Normal BMI was defined as between the 5th and 85th percentile, as per the Center for Disease Control growth charts [26]. Body volume was determined using air displacement plethysmography via the BOD POD (COSMED USA Inc., Concord, CA, USA), and fat-mass and fat-free mass were estimated using the Lohman body density equation [27]. Prior to this assessment, participants were instructed to fast for two hours.

### 2.2. Experimental Design

Three VAS instruments assessing motivation-to-eat were used in the present study: traditional paper-based (VAS_paper_), digital with images (VAS_images_), and digital with no images (VAS_no-images_). The digital software application Express VAS (Express VAS, Toronto, Canada) is a novel image-based software program designed to assess motivation-to-eat and subjective thirst in the pediatric age range. On one weekend morning, participants arrived at the laboratory between 1100 and 1200 h, 3 h after consuming a standardized breakfast provided by our laboratory (a strawberry cereal bar (130 kcal; Selections, Montreal, QC, Canada), a pear fruit cup (90 kcal, Selection, Montreal, QC, Canada), and an orange juice box (100 kcal; Minute Maid, Toronto, ON, Canada)). Immediately after baseline measurements, participants consumed a 147 kcal plain yogurt snack (233.9 g, 3% milk fat; Astro, Lactalis Canada, Toronto, ON, Canada) along with 350 mL of water. Participants were instructed to consume the yogurt snack and water within 5 min in order to complete the next VAS measurement at the 5-min timepoint. Thirty-five minutes after baseline, participants consumed an ad libitum pizza lunch. All three VAS instruments were completed in a random order at five time points: 0 (baseline), 5 (immediately post-yogurt treatment), 20, 35 (immediately pre-ad libitum pizza lunch), and 65 min (immediately post-ad libitum lunch).

### 2.3. Visual Analogue Scales

**Figure 1:**
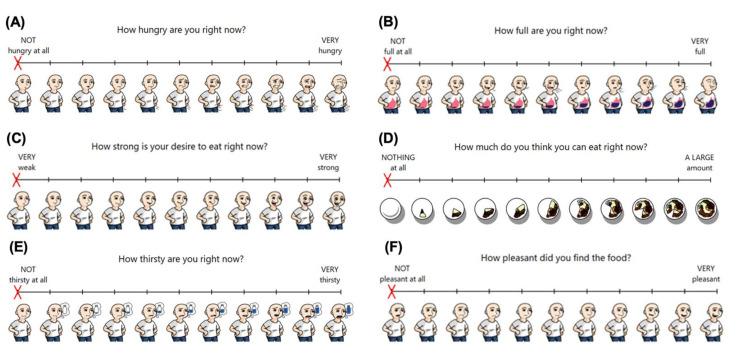
VAS_images_ schematics: (**A**) Hunger, (**B**) Fullness, (**C**) Desire To Eat (DTE), (**D**) Prospective Food Consumption (PFC), (**E**) Thirst, and (**F**) Pleasantness.

Each of the three VAS instruments (VAS_paper_, VAS_images_, and VAS_no-images_) included four domains: (A) hunger, (B) fullness, (C) desire to eat (DTE), and (D) prospective food consumption (PFC) [2,21] (Figure 1A–D). A subjective average appetite (AA) score was calculated using the following formula [21]:

AA = (DTE + Hunger + (100 − Fullness) + PFC)/4

To evaluate subjective thirst during the session, a VAS question “How thirsty do you feel?” was also measured (Figure 1E). Pleasantness was assessed following the yogurt treatment and ad libitum pizza meal using the VAS question “How pleasant did you find the food?” (Figure 1F). The digital VASs (VAS_images_ and VAS_no-images_) were developed as a Windows application and were administered using a Dell Latitude 5290 two-in-one tablet (Dell Canada, North York, ON, Canada). Each digital VAS had ten markings along the line dividing it into equal segments. Each question in VAS_images_ had ten images along the VAS continuum that corresponded to each of the ten markings (Figure 1). For example, the ‘not full at all’ anchor on the VAS_images_ fullness domain depicted an animated character with an empty stomach, whereas the ‘very full’ anchor depicted the identical animated character but with a full stomach (i.e., the stomach fills progressively as the continuum increases) (Figure 1B). VAS_no-images_ was identical to VAS_images_ as depicted in Figure 1 but without the images. Each digital VAS was a 100 unit line (190 mm), while the traditional VAS_paper_ instrument uses a standard 100 mm line [16]. Each question was provided on a separate page (VAS_paper_) or screen (VAS_images_ and VAS_no-images_). With each VAS instrument, participants received standardized instructions to place a mark on the line to indicate their hunger, fullness, DTE, PFC, thirst, and pleasantness. Markings on the digital VAS were automatically recorded within the application. Markings on VAS_paper_ were measured by two independent raters; if the difference between the raters was greater than 0.5 mm, a third independent rater conducted a measurement and the mean VAS score using the two closest measurements was calculated and used in all subsequent statistical analyses.

### 2.4. Ad Libitum Food Intake

Immediately after filling out the VAS instruments at 35 min, participants were given an ad libitum pizza lunch with instructions to eat until they were comfortably full. Participants were served a tray containing three pizzas (Dr. Oetker Canada Ltd., Mississauga, ON, Canada). Cheese or pepperoni pizzas were cut into quarters based on participant preference; each tray contained two pizzas of their first choice and one of their second choice. Both types of pizzas were small and round and were of similar nutritional composition (12.5 cm diameter, ~85 g, and ~190 kcal). If more than 50% of the last tray was eaten, participants were given another tray of pizza in 10-min increments. Participants were also provided with ad libitum water using 500 mL bottled water (Nestle Pure Life, Guelph, ON, Canada). Participants consumed their meal in individual cubicles to limit distractions from other research participants. FI was determined by weighing the meal before and after serving; the net weight (g) of the test meal was converted to kcal based on the nutrition information provided by the manufacturer. The participants were not aware that their pizza lunch was being weighed before or after consumption. Total FI was based on the kcal consumed at the ad libitum lunch. Each 500 mL water bottle was weighed (g) before and after to determine water intake.

### 2.5. Data Analysis

Participant characteristics are presented as means and standard error of the mean (SEM). To allow for appropriate comparison between paper and digital VAS instruments, measurements are reported in units, where each unit is 1 mm using VAS_paper_, and each unit is 1/100th of the length of the line for VAS_images_ and VAS_no-images_.

To evaluate agreement between the traditional VAS_paper_ and the digital versions (VAS_images_, VAS_no-images_), Bland–Altman plots, intraclass correlation coefficients (ICC), and analysis of variance (ANOVA) were performed. Bland–Altman plots with 95% limits of agreement (LOA = 1.96 SD) were used to graphically assess and calculate, respectively, the agreement between instruments for AA immediately before the ad libitum lunch (35 min) [28]. ICCs were calculated for the four motivation-to-eat domains (hunger, fullness, DTE, PFC), as well as AA and thirst at each time-point (0, 5, 20, 35, 65 min). ICC values of <0.50 indicate poor agreement, 0.50–0.75 moderate agreement, 0.75–0.90 good agreement, and >0.90 excellent agreement [29]. Bland–Altman plots and ICCs were used to compare: VAS_paper_ and VAS_images_, VAS_paper_ and VAS_no-images_, and VAS_images_ and VAS_no-images_. Lastly, the incremental area under the curve (iAUC) of each motivation-to-eat domain, AA, and thirst were calculated using the trapezoid method [30], and one-way repeated measures ANOVA was performed to assess the agreement between the three VAS instruments. A two-way repeated measures mixed model ANOVA was performed to assess the differences between motivation-to-eat scores and thirst at each time-point. A Tukey post-hoc test was used to evaluate the effect of time, instrument, and/or time by instrument interactions for each domain from the one-way and two-way ANOVAs.

To evaluate the predictive validity of each VAS instrument for subsequent FI, two-tailed Pearson correlations were performed between FI and hunger, fullness, DTE, PFC, thirst, and pleasantness at each time-point (0, 5, 20, 35, and 65 min).

IBM Statistics SPSS 26 (IBM, New York, NY, USA) was used for all Bland–Altman plots, ICCs, and Pearson correlations. Bland–Altman plots were created using GraphPad Prism version 9.0 (GraphPad Software, La Jolla, CA, USA). SAS version 9.3 (SAS Institute Inc., Carey, NC, USA) was used for one-way and two-way repeated measures ANOVA with the Tukey post-hoc test when significance was observed. The level of statistical significance was set at *p* < 0.05.

## 3. Results

### 3.1. Participant Characteristics

Seventeen children and adolescents (*n* = 9 girls, *n* = 8 boys) participated in the study. Baseline characteristics are summarized in Table 1.

### 3.2. Agreement between Visual Analogue Scale Instruments

#### 3.2.1. Bland–Altman Plots

**Figure 2:**
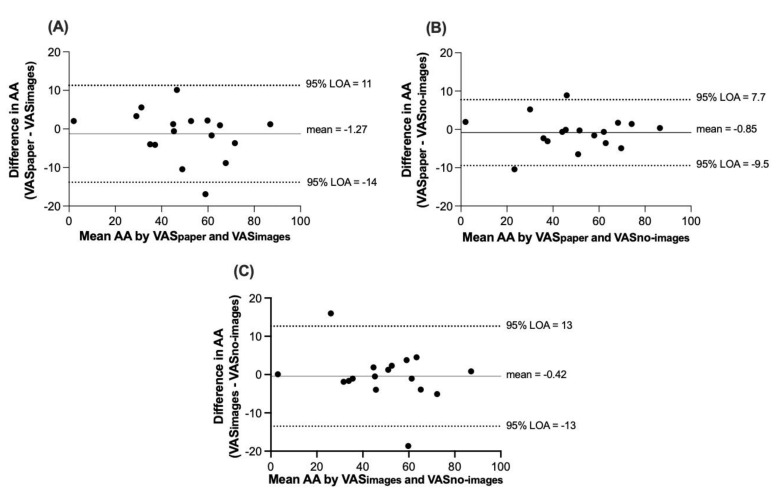
(**A**) Bland–Altman plots (95% limits of agreement) of the difference in VAS_paper_ and VAS_images_ (units) versus the mean of VAS_paper_ and VAS_images_ for average appetite (AA) immediately before the ad libitum lunch (35 min). The same comparison is made for (**B**) VAS_paper_ and VAS_no-images_ and for (**C**) VAS_images_ and VAS_no-images_. *n* = 17.

The mean difference between VAS_paper_ and VAS_images_ for AA immediately before the ad libitum lunch (35 min) was −1.27 units (95% LOA: −14 to 11), between VAS_paper_ and VAS_no-images_ was −0.85 units (95% LOA: −9.5 to 7.7), and between VAS_images_ and VAS_no-images_ was −0.42 units (95% LOA: −13 to 13) (Figure 2A–C).

#### 3.2.2. Intraclass Correlation Coefficients

ICCs between VAS_paper_ and VAS_no-images_, VAS_paper_ and VAS_images_, and VAS_images_ and VAS_no-images_ all revealed good to excellent agreement for each domain at each time-point (Table 2).

#### 3.2.3. Analysis of Variance

One-way repeated measures ANOVA (using calculated iAUCs) found no differences between the three instruments for any motivation-to-eat domain, AA, or thirst (*p* = 0.73–0.97). One-way ANOVA found no differences among the three instruments for pleasantness of the snack (F(1.211, 18.160) = 0.67, *p* = 0.45) or pizza lunch (F(2, 28) = 0.75, *p* = 0.480).

**Figure 3:**
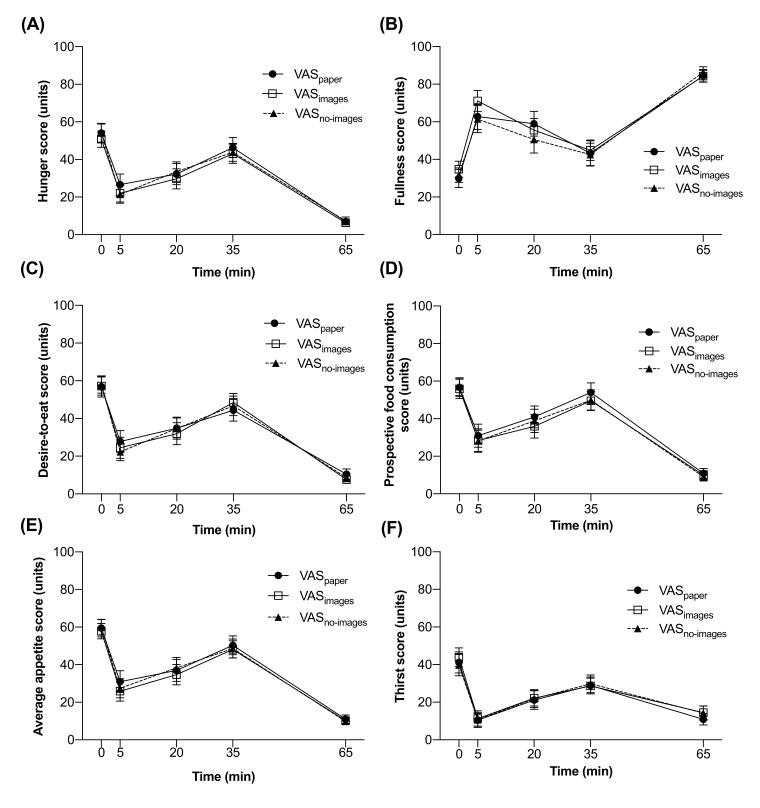
Agreement between three VAS instruments at five time points for: (**A**) hunger, (**B**) fullness, (**C**) desire-to-eat, (**D**) prospective food consumption, (**E**) average appetite, and (**F**) thirst. All values are means ± SEM, *n* = 17 (*n* = 9 girls, *n* = 8 boys). Two-way ANOVA revealed no instrument by time interaction (*p* > 0.58), and no main effect of instrument (*p* > 0.31), but a main effect of time (*p* < 0.001).

Two-way repeated measures ANOVA found no instrument × time interactions for any of the four motivation-to-eat domains, AA, or thirst (*p* > 0.88). There were no main effects of instrument type for hunger (*p* = 0.31), fullness (*p* = 0.34), DTE (*p* = 0.89), PFC (*p* = 0.33), AA (*p* = 0.41), or thirst (*p* = 0.73). A main effect for time (*p* < 0.001) was found for all four domains (Figure 3A–D), AA (Figure 3E), and thirst (Figure 3F).

### 3.3. Predictive Validity of Visual Analogue Scale Instruments

Immediately before the ad libitum lunch (35 min), statistically significant correlations were found between FI and hunger, DTE, PFC, and AA for all three instruments (VAS_paper_, VAS_images_, or VAS_no-images_) (Table 3). Thirst did not correlate with FI at any of the measurement time-points. Additionally, there was no significant association between subjective thirst and water intake, except when using VAS_no-images_ at baseline (r = 0.49, *p* = 0.046). Pleasantness of the yogurt snack (5 min) did not significantly correlate with FI. Furthermore, pleasantness of the pizza (65 min) was not associated with FI when measured by VAS_no-images_ or VAS_images_; however, a significant association was observed with VAS_paper_ following the ad libitum meal at 65 min (Table 3).

## 4. Discussion

The present study was designed to assess agreement between the traditional paper-based and a novel digital motivation-to-eat VAS (with and without images), and also to assess the predictive validity of a novel digital VAS for subsequent FI in children and adolescents aged 9–14 years. There was good agreement between the three instruments across all domains and time-points, suggesting that the novel digital VAS (with and without images) can be used interchangeably with paper-based versions in the pediatric population. All three test instruments showed good predictive validity of hunger, DTE, PFC, and AA for subsequent FI. An exception to these findings was observed in the fullness domain where VAS_images_ and VAS_no-images_ showed no significant correlations between fullness and FI at any time point, and VAS_paper_ only had a significant correlation at 20 min. Previous work in adults has reported that fullness was not predictive of FI [31,32]; our findings were similar in that fullness was not a strong contributing factor for predicting subsequent FI in children and adolescents. However, more research is warranted given the relatively small number of studies that have evaluated the association between the individual motivation-to-eat domains and FI.

Previous research has reported agreement between paper-based and digital VAS instruments, such as the Electronic Appetite Rating System (EARS), ProDiary^©^, and APPetite; however, there is a consensus that despite the good agreement they are not interchangeable [13,14,15,16,17,18,19]. For example, mean appetite ratings showed high variability when comparing paper to digital VAS measures [14,15], perhaps in part due to end-of line effects whereby participants avoid making ratings at the extremes of the line on the digital VAS [16] and EARS [13]. Indeed, the current study found VAS_paper_ measures were consistently higher than the two digital variants (VAS_images_, VAS_no-images_); however, these findings were not significant (as shown by good to excellent ICCs between instruments for all domains at all time-points, and no differences between iAUC for any of the domains between instruments) so it does not appear as though end-of line effects significantly impacted participant ratings in the present study. This may be related to the fact that end-of-line effects have been observed in digital VASs with smaller scales (e.g., 52 mm), while the digital VAS lines in the present study were 190 mm. Of note, previous research has suggested that a sample size of 12 participants is sufficient to detect within-subject differences between VAS methods [12]; therefore, with *n* = 17 the present study was adequately powered to detect differences among test instruments.

Regarding the use of images in motivation-to-eat VASs, a previous study in healthy adults asked how many portions they could eat of 10 different photographed foods and reported no differences between the image-based and traditional VAS [32]. More specific to youth, other subjective appetite rating systems have featured pictures and visual cues to model levels of appetite for children [22,23,24,25]. Although the children in these studies were younger than those in the present study (4–8 years vs. 9–14 years), it was hypothesized that a greater understanding of subjective appetite would be fostered through images. Two studies found that children were able to use a category-based system to accurately describe imagined eating situations [22,24]. For example, children aged 4–5 years used a slider to fill an animated character’s abdomen to reflect fullness [24]. One study reported differences in ratings before and after an ad libitum meal when using a categorical appetite system [22]. Finally, a recent study that evaluated a picture-based appetite assessment tool to detect hunger and fullness cues in children aged 4–10 years found that the tool was able to detect expected changes in appetite sensations; the tool also had good agreement with the traditional paper-based VAS instrument [25].

The present study is the first to report the predictive validity of a digital VAS for subsequent FI in older children and adolescents. Previous research reported no associations between motivation-to-eat scores measured using the paper-based VAS and subsequent FI during an ad libitum lunch in young boys, possibly due to a combination of the low sample size and inclusion of children with overweight and obesity [21]. Further, a recent systematic literature review determined that subjective appetite ratings were not predictive of FI in 52.4% of studies conducted in children (51.3% of studies conducted in adults) [33]. However, the studies included in this review were not VAS validation studies, and thus their findings may not be fully applicable to the findings observed in the present study. While the present study found measurements of thirst to agree across the three VAS instruments (VAS_paper_, VAS_images_, and VAS_no-images_), the predictive validity of VAS measurements for water intake were not significant. The validity of thirst as measured by a VAS and its association with water intake during an ad libitum meal is not well understood. There have been no other studies examining the validity of thirst ratings in healthy children. In adults, several studies have evaluated the use of a VAS for thirst ratings. One study found that 24 h of water deprivation resulted in significant increases in subjective ratings of thirst; however, after participants were able to drink water, ad libitum thirst ratings declined rapidly, followed by a gradual decrease [34]. These findings may suggest that VAS ratings for thirst have limited sensitivity when hydration levels are normal. Furthermore, one review concluded that thirst ratings cannot be used to predict fluid consumed ad libitum [35].

A source of strength for the present study is that motivation-to-eat scores were compared to subsequent FI to determine their predictive validity. Of 462 studies in a recent systematic review that measured both appetite ratings and FI, 97.3% did not report directly on such a relationship [33]. Limitations in this study include that there have been concerns that repeated measurements of appetite by VASs may influence FI by way of continually cuing one’s state of hunger or fullness [5,36]. This is an important methodological consideration, as participants in the current study completed three VAS instruments at five time-points. However, a recent study showed that completion of multiple VASs at a time does not have any significant bearing on subsequent FI [5]. In the present study, the VAS instruments were presented in a random order for each participant at every time-point to reduce the risk of instrument order effects. However, it is possible there were carryover effects of the previous test instrument. Another limitation of this study is that preloads of different quantities were not considered. As such, the sensitivity of VAS_images_ and VAS_no-images_ as compared to VAS_paper_ is unknown; future studies investigating the sensitivity and utility of digital VASs are needed in the pediatric population [16]. Further, the controlled laboratory nature of this study may not translate to a free-living environment. This research is also specific to children and adolescents between the ages of 9–14 years and of normal body weight. Future research investigating the predictive validity of VAS_images_ and VAS_no-images_ in other populations and settings is warranted. Finally, future studies may consider evaluating post-prandial appetite over a longer timeframe, and should consider stratification by sex and other sociodemographic variables.

## 5. Conclusions

The results of the present study suggest that a novel image-based digital VAS evaluating motivation-to-eat is interchangeable with the traditional paper-based VAS, and provides good predictive validity of next-meal FI in 9–14-year-old normal weight children and adolescents.

## Figures and Tables

**Table 1:** Baseline Characteristics (*n* = 17; 8 boys, 9 girls).

Variable	Means ± SEM	Range
Age (years)	11.5 ± 0.3	9–14
Height (cm)	154.5 ± 2.1	140.5–169.0
Body mass (kg)	44.5 ± 2.1	29.9–62.5
BMI percentile *	50.1 ± 6.6	6–85
Fat mass ^1^ (%)	25.9 ± 1.6	12.5–38.7
Fat-free mass ^1^ (%)	74.1 ± 6.7	61.3–87.5

Abbreviations: BMI, body mass index; Standard Error of the Mean, SEM. * BMI percentiles were calculated according to the Centers for Disease Control growth charts [26]. ^1^ Body composition measures (i.e., fat mass and fat-free mass) were estimated using the Bod Pod and age-specific density equations [27].

**Table 2:** ICCs evaluating agreement between the three VAS instruments.

Ratings Instruments		0 min	5 min	20 min	35 min	65 min
Hunger	VAS_paper_ vs. VAS_no-images_	0.95 (0.85–0.98)	0.85 (0.60–0.95)	0.90 (0.72–0.96)	0.94 (0.83–0.98)	0.92 (0.78–0.97)
	VAS_paper_ vs. VAS_images_	0.92 (0.77–0.97)	0.94 (0.82–0.98)	0.90 (0.74–0.97)	0.94 (0.83–0.98)	0.95 (0.86–0.98)
	VAS_images_ vs. VAS_no-images_	0.97 (0.90–0.99)	0.94 (0.82–0.98)	0.96 (0.88–0.99)	0.97 (0.91–0.99)	0.97 (0.91–0.99)
Fullness	VAS_paper_ vs. VAS_no-images_	0.94 (0.83–0.98)	0.86 (0.60–0.95)	0.89 (0.70–0.96)	0.98 (0.95–0.99)	0.94 (0.83–0.98)
	VAS_paper_ vs. VAS_images_	0.89 (0.69–0.96)	0.80 (0.47–0.93)	0.77 (0.32–0.92)	0.90 (0.72–0.96)	0.94 (0.83–0.98)
	VAS_images_ vs. VAS_no-images_	0.89 (0.71–0.96)	0.80 (0.45–0.93)	0.86 (0.63–0.95)	0.91 (0.75–0.97)	0.97 (0.89–0.99)
DTE	VAS_paper_ vs. VAS_no-images_	0.91 (0.75–0.97)	0.90 (0.70–0.96)	0.91 (0.75–0.97)	0.92 (0.77–0.97)	0.72 (0.25–0.90)
	VAS_paper_ vs. VAS_images_	0.83 (0.52–0.94)	0.94 (0.83–0.98)	0.88 (0.67–0.96)	0.89 (0.69–0.96)	0.72 (0.25–0.90)
	VAS_images_ vs. VAS_no-images_	0.89 (0.69–0.96)	0.93 (0.82–0.98)	0.94 (0.82–0.98)	0.95 (0.82–0.99)	0.96 (0.90–0.99)
PFC	VAS_paper_ vs. VAS_no-images_	0.98 (0.94–0.99)	0.98 (0.95–0.995)	0.98 (0.96–0.99)	0.95 (0.86–0.98)	0.92 (0.79–0.97)
	VAS_paper_ vs. VAS_images_	0.95 (0.86–0.98)	0.94 (0.84–0.98)	0.95 (0.84–0.984)	0.97 (0.85–0.99)	0.88 (0.67–0.96)
	VAS_images_ vs. VAS_no-images_	0.97 (0.91–0.99)	0.95 (0.85–0.98)	0.96 (0.89–0.99)	0.97 (0.91–0.99)	0.90 (0.72–0.96)
AA	VAS_paper_ vs. VAS_no-images_	0.97 (0.92–0.99)	0.97 (0.91–0.99)	0.98 (0.95–0.99)	0.99 (0.97–0.996)	0.94 (0.83–0.98)
	VAS_paper_ vs. VAS_images_	0.94 (0.85–0.98)	0.95 (0.83–0.98)	0.93 (0.79–0.97)	0.97 (0.93–0.99)	0.92 (0.78–0.97)
	VAS_images_ vs. VAS_no-images_	0.98 (0.94–0.99)	0.94 (0.85–0.98)	0.94 (0.85–0.98)	0.97 (0.93–0.99)	0.98 (0.94–0.99)
Thirst	VAS_paper_ vs. VAS_no-images_	0.96 (0.90–0.99)	0.95 (0.87–0.98)	0.96 (0.90–0.99)	0.96 (0.88–0.98)	0.93 (0.78–0.97)
	VAS_paper_ vs. VAS_images_	0.93 (0.82–0.98)	0.94 (0.84–0.98)	0.95 (0.85–0.98)	0.94 (0.82–0.98)	0.80 (0.46–0.93)
	VAS_images_ vs. VAS_no-images_	0.94 (0.83–0.98)	0.91 (0.75–0.97)	0.97 (0.93–0.99)	0.97 (0.91–0.99)	0.90 (0.71–0.96)

Abbreviations: Average appetite, AA; Desire to eat, DTE; Intraclass correlation coefficient, ICC; Prospective food consumption, PFC. Results are expressed as mean ICC (95% confidence interval).

**Table 3:** Pearson correlations evaluating predictive validity of the three VAS instruments on next-meal food intake.

Instrument	Domain	0 min	5 min	20 min	35 min	65 min
VAS_paper_	Hunger	0.47	0.51 *	0.64 **	0.49 *	−0.004
	Fullness	−0.20	−0.47	−0.64 **	−0.21	0.26
	DTE	0.48 *	0.57 *	0.63 **	0.61 **	−0.13
	PFC	0.64 **	0.75 ***	0.75 ***	0.74 ***	−0.16
	AA	0.50 *	0.63 **	0.74 ***	0.56 *	−0.18
	Thirst	0.19	−0.06	−0.01	−0.32	−0.16
	Pleasantness ^1^	-	0.28	-	-	0.60 *
VAS_no-images_	Hunger	0.49 *	0.74 ***	0.75 ***	0.61 **	0.01
	Fullness	−0.33	−0.30	−0.37	−0.26	0.38
	DTE	0.42	0.82 ***	0.69 **	0.71 ***	0.04
	PFC	0.70 **	0.80 ***	0.74 ***	0.71 ***	−0.16
	AA	0.54 *	0.68 **	0.67 **	0.60 *	−0.18
	Thirst	0.21	−0.12	−0.12	−0.34	−0.32
	Pleasantness ^1^	-	0.32	-	-	0.23
VAS_images_	Hunger	0.50 *	0.71 ***	0.76 ***	0.53 *	0.04
	Fullness	−0.17	−0.45	−0.41	−0.41	0.32
	DTE	0.53 *	0.72 ***	0.75 ***	0.65 **	0.13
	PFC	0.63 **	0.74 ***	0.77 ***	0.76 ***	−0.08
	AA	0.53 *	0.69 **	0.73 ***	0.63 **	−0.12
	Thirst	0.16	−0.15	−0.03	−0.19	−0.34
	Pleasantness ^1^	-	0.20	-	-	0.29

Abbreviations: AA, average appetite; DTE, desire-to-eat; PFC, prospective food consumption; VAS, visual analogue scales. All values are correlation coefficients and represented by different levels of statistical significance, * *p* < 0.05; ** *p* < 0.01; *** *p* < 0.001, *n* = 17. ^1^ Pleasantness rating at 5 min represents response to yogurt snack; 65 min represents response to pizza meal.

## Data Availability

The data presented in this study are available on request from the corresponding author.
